# Barriers and enablers to addressing smoking, nutrition, alcohol consumption, physical activity and gestational weight gain (SNAP-W) as part of antenatal care: A mixed methods systematic review

**DOI:** 10.1186/s43058-024-00655-z

**Published:** 2024-10-09

**Authors:** Sophie Dilworth, Emma Doherty, Carly Mallise, Milly Licata, Jenna Hollis, Olivia Wynne, Cassandra Lane, Luke Wolfenden, John Wiggers, Melanie Kingsland

**Affiliations:** 1 Population Health, Hunter New England Local Health District, Wallsend, NSW 2287 Australia; 2 School of Medicine and Public Health, College of Health, Medicine and Wellbeing, The University of Newcastle, Callaghan, NSW 2308 Australia; 3 Population Health Research Group, Hunter Medical Research Institute, New Lambton Heights, New Lambton Heights, NSW 2305 Australia; 4 Nursing and Midwifery Research Centre, Hunter New England Local Health District, Newcastle, NSW 2300 Australia; 5 Clinical Research Design, IT and Statistical Support, Hunter Medical Research Institute, New Lambton Heights, New Lambton Heights, NSW 2305 Australia

**Keywords:** Preventive, Pregnancy, Smoking, Alcohol, Weight, Nutrition, Physical activity, Barriers, Theoretical domains framework, Systematic review

## Abstract

**Background:**

International clinical guidelines recommend that smoking, nutrition, alcohol consumption, physical activity and gestational weight gain (SNAP-W) be addressed as part of routine antenatal care throughout pregnancy. However, guideline recommendations are poorly implemented, and few antenatal care recipients routinely receive the recommended care. There is a need to establish the determinants (barriers and enablers) to care delivery to inform strategies to improve implementation. This systematic review aimed to synthesize qualitative and quantitative evidence of the barriers and enablers to the routine delivery of antenatal care targeting SNAP-W health risks.

**Methods:**

A systematic review was conducted following Preferred Reporting Items for Systematic Reviews and Meta-Analyses guidelines. Seven databases were searched for relevant studies published between January 2001 and November 2023. Study findings were coded and analysed according to the domains of the Theoretical Domains Framework (TDF).

**Results:**

Forty-nine studies were included in the review, 27 qualitative studies and 22 quantitative studies. The studies were conducted in 14 countries. Data were collected from 7146 antenatal care providers (midwives, Aboriginal health workers, obstetricians, medical officers, general practitioners) and 352 barriers and enablers were identified. Across all SNAP-W health risk and antenatal care provider groups, the predominant TDF domain was ‘environmental context and resources’, identified in 96% of studies. Barriers within this domain included insufficient time, limited access to and quality of resources, and limited organisational supports. ‘Beliefs about consequences’ was the second most common TDF domain, reported in 67% of studies, particularly studies of care related to alcohol use, nutrition/ physical activity/ gestational weight gain and those involving midwives, multidisciplinary practitioners and general practitioners. ‘Optimism’ was the second most common TDF domain for studies of smoking-related care and involving obstetricians, gynaecologists, and other mixed medical professions.

**Conclusions:**

It is critical that determinants related to environmental context and resources including time, resources and organisational supports are considered in the development of strategies to support the implementation of recommended antenatal care related to SNAP-W risks. Strategies addressing clinician beliefs about consequences and optimism may also be needed to support the implementation of care related to specific health behaviours and by specific antenatal care provider groups.

**Registration:**

The review protocol was prospectively registered with Prospero: CRD42022353084; 22 October 2022.

**Supplementary Information:**

The online version contains supplementary material available at 10.1186/s43058-024-00655-z.

Contributions to the literature • This review synthesises barriers and enablers to the routine delivery of SNAP-W care during pregnancy and codes them into the Theoretical Domains Framework (TDF). It is the first review with this focus to use a theoretically informed approach to synthesis.

• The inclusion of qualitative and quantitative studies provides a deeper understanding of SNAP-W antenatal care delivery determinants.

• Barriers associated with the environmental context and resources are the most commonly reported barriers to antenatal care provision related to SNAP-W.

## Background

Modifiable risk factors during pregnancy can have significant implications for pregnant people and their babies [1]. Tobacco smoking, suboptimal nutrition, alcohol consumption, physical inactivity and gestational weight gain outside of recommended ranges (SNAP-W) are associated with an increased risk of pregnancy complications and poor obstetric outcomes, including spontaneous abortion, small or large for gestational age, preterm birth, and need for neonatal intensive care [2–6]. Further negative impacts include poor infant and child outcomes, such as developmental delay and obesity [2, 3, 7–9]. Clustering of these modifiable risk factors during pregnancy is also well established and can increase such risks through cumulative effects [10–12]. Internationally, it is estimated that 10% of pregnant people smoke tobacco [13–15], 10% consume alcohol [16], and 68% gain weight outside of recommended ranges [1, 2, 17, 18]. However, these rates vary considerably, with much higher reported prevalence in some countries and population groups [1].

Evidence-based international clinical guidelines recommend that SNAP-W health risks, be addressed as part of routine antenatal care at initial appointments and throughout pregnancy [1, 19–21]. Such care is recommended to include assessment of risk status using a validated or objective measure; discussion of the risk factor recommendations and potential harms; and offer of further evidence-based support, such as referral to services for counselling, or provision of pharmaceutical support (such as nicotine replacement therapy), if required. However, these clinical guidelines are poorly implemented, with international evidence showing few antenatal care recipients routinely receive the recommended care [22–26]. Unless routinely implemented, the intended benefits of antenatal clinical guidelines in supporting healthy pregnancies will not be fully realised.

To support improvements to the implementation of guideline recommended care, an understanding of the determinants to implementation from the perspective of antenatal care professionals is required [27, 28]. Studies of antenatal care provision have shown that implementation strategies designed to target care-delivery barriers reported by health professionals are effective in supporting the delivery of recommended care [29–32]. Identification of barriers and enablers is a recommended step in the design of implementation and health care quality improvement strategies [27, 33]. The use of theoretical frameworks to inform this process increases effectiveness of implementation strategies by targeting behavioural determinants and underlying mechanisms required to change healthcare professionals’ behaviours [28].

Despite the importance, no reviews have synthesised evidence on barriers and enablers for SNAP-W risk behaviours for antenatal health professionals using a theoretical framework. Existing reviews have focused on individual health risk areas [34–36], been limited to synthesis of barriers [35], included only qualitative evidence, or not used a theoretical framework [34, 35].

## Objectives

The objectives of our review were to systematically review and synthesise the literature for qualitative and quantitative evidence according to the Theoretical Domains Framework (TDF) [37] to: 1) describe the barriers and enablers reported by health professionals in the delivery of antenatal SNAP-W care provision; and 2) compare barriers and enablers by health risk and healthcare profession.

## Methods

We followed the Preferred Reporting Items for Systematic Reviews and Meta‐Analyses (PRISMA) Guidelines [38] when conducting this review (see Additional file 1). The review protocol was prospectively registered with Prospero: CRD42022353084; 22 October 2022.

### Searches

The search strategy was developed in consultation with research librarians (See Additional file 2) and run across seven electronic bibliographic databases: MEDLINE, EMBASE, PsycINFO, Maternity and Infant Care, Scopus, CINAHL and Cochrane Library. Reference lists of included studies and relevant reviews were also screened. The search was limited to articles published in the past 22 years (2001). Studies published 22 or more years ago were excluded due to the likelihood of significant changes in policy and guideline recommendations. The country of origin was not restricted. Only studies published in English were eligible for consideration, due to lack of resources.

Search results were uploaded to Covidence for screening, data extraction, and quality assessment [39]. Following removal of duplicates, study titles and abstracts, then full texts were screened for eligibility by two independent reviewers (SD and either ED, OW, CM). Discrepancies regarding study eligibility were resolved through discussion and consensus between the three reviewers (SD, ED and either OW or CM). Where there was insufficient information to determine study eligibility, a reviewer contacted the original study author(s) for clarification.

### Study inclusion and exclusion criteria

Studies were included if they explored antenatal care provider’s perceived or experienced barriers and/or enablers to antenatal care related to the provision of SNAP-W care (assessment, advice, assistance) during pregnancy. Antenatal care was defined as pregnancy/ antenatal/ prenatal care from the time when pregnancy is confirmed to birth, delivered in any health care setting including, hospital outpatient clinics, primary healthcare, or community care settings. Barriers were defined as anything that impeded or obstructed the delivery of care and enablers as anything that eased or promoted the delivery of care [40]. Barriers/enablers had to be reported as an aim/ objective of the study or in the outcomes, not inferred by discussion. Antenatal care providers were health professionals involved in routine antenatal care as their primary specialty, such as midwives, Aboriginal health workers, obstetricians, and medical officers working in maternity services/ specialty areas, including general practitioners/ family physicians. Only primary qualitative, quantitative, and mixed method studies were included. Studies were excluded if the outcomes reported the perspective of health professionals not involved in the routine delivery of antenatal care, patients or health risk behaviours that could not be considered separately from the topic of the review or were focused on a subset of women and barriers related to their specific needs and care, e.g., Gestational Diabetes. Studies evaluating barriers/enablers to antenatal care providers participation in the implementation of a specific intervention/ program, were also excluded.

### Study quality assessment

The Mixed Methods Appraisal Tool (MMAT) [41, 42] was used to appraise the methodological quality of included studies. Two review authors independently applied the MMAT to each study (MK and JH, SD and CL), with disagreements resolved through discussion with a third review author as needed (SD, MK). The MMAT was chosen because it can be applied to various study designs, including, quantitative observational, qualitative, and mixed-methods studies [43, 44]. The quality appraisal was not used to exclude studies.

### Data extraction strategy

Standardised data extraction forms for qualitative and quantitative research were developed and piloted before use. Two reviewers independently carried out data extraction of all included studies (SD and ML, ED, CM), and reached agreement in consultation with three reviewers (SD, ED, MK). Data items included the following: article citation, country, theoretical approach, aim of study, SNAP-W health risk behaviour/s, study design, data collection method, population, practice setting, sample size, presentation of results and main findings/ illustrations of findings. Where studies reported on the prevalence of all barriers/enablers included in data collection tools irrespective of the proportion of participants that reported being influenced by the barrier/enablers a nominal cut point of 30% was applied to distinguish between determinants that were reported by a substantial proportion of participants rather than just examined. Following data extraction and quality assessment within Covidence, data were exported to Microsoft Excel to facilitate synthesis.

### Data synthesis and presentation

The protocolised parallel integrated approach to data synthesis was not possible due to heterogeneity in the reported quantitative data. As per protocol, the synthesis moved to a convergent approach that coded the quantitative and qulitative data sets against a pre-determined framework [45, 46]. Extracted quantitative data were ‘qualitized’ [45–47], a process that converts quantitative data into ‘textual descriptions’ to allow integration with qualitative data [45–47]. This method is recommended as it is less error-prone than attributing numerical values to qualitative data [48]. Survey items and response options were considered as textual descriptions of barriers/enablers. Where studies reported barriers/enablers as aggregated categories/ domains the category label was considered as the textual description. These textual descriptions were pooled with the data extracted directly from qualitative studies [45, 46]. To bring the data together, all extracted data were coded against a pre-determined framework [49]: the TDF [28, 50], see Additional file 3. Two review authors (MK and ED) independently coded the extracted barriers and enablers to the TDF. To ensure consistency and ‘fit’ within the framework, all coding was reviewed, discussed, and agreed by three review authors (MK, ED, SD). The synthesis presents the cumulative frequency of barrier and enablers coded for each domain (i.e., the number of times a domain was coded overall, including repeated coding from single studies). The number of studies that identified each domain at least once was also reported to reduce the risk of confirmatory bias, from studies that focussed only on a single or limited number of domains.

During synthesis, exact quotes and phrasing from primary studies were not modified to accurately report on the primary study findings. It is noted that language in the primary studies may not reflect inclusivity in gender identity. Elsewhere in the review, inclusive language has been used in recognition of the different gender identities of birthing parents.

## Results and discussion

### Search results

**Fig. 1:**
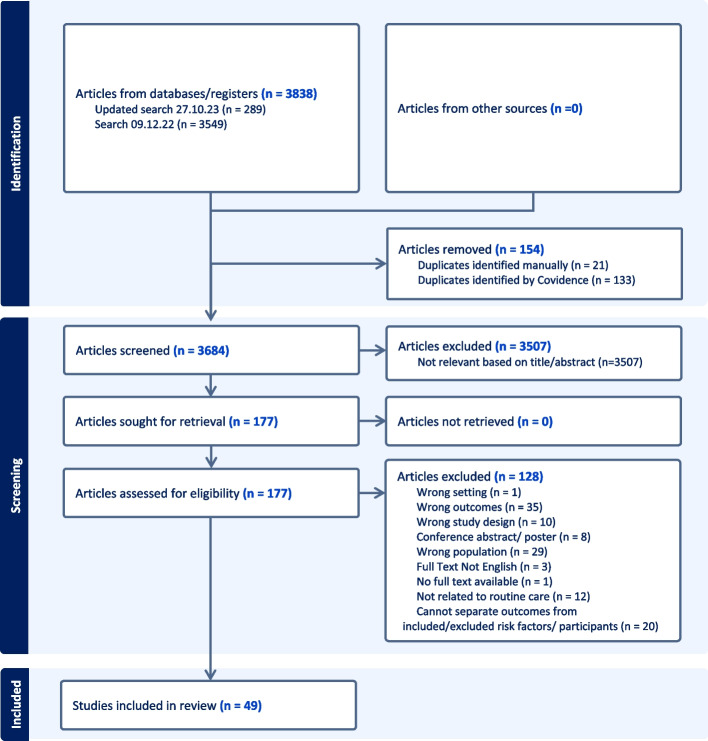
PRISMA flow diagram

The search strategy was run up to October 2023 and identified 3684 unique articles. Following title and abstract screening, 177 full text articles were assessed for eligibility, resulting in 49 studies that were included in the review (Fig. 1).

### Study characteristics

The review includes 49 studies with data collected from 7146 antenatal care providers. Included studies were published between 2001 and 2023 and conducted across 14 countries, predominately the United States (*n* = 14), Australia (*n* = 12), and the United Kingdom (*n* = 7). Regarding study design, 27 studies used qualitative designs, 20 studies used a cross sectional survey, and two studies reported the use of a mixed methods design. However, the two mixed methods studies reported findings generated via a survey only and have been considered as quantitative studies in this review. The antenatal care providers included in studies were primarily midwives (*n* = 22), followed by multidisciplinary groups (*n* = 12), obstetrician/gynaecologists (*n* = 6), general practitioners (*n* = 5), and mixed/unspecified medical practitioners (*n* = 5). The most common SNAP-W health risk examined in studies was smoking (*n* = 22), followed by nutrition/ physical activity/ gestational weight gain (*n* = 18) and alcohol consumption (*n* = 9). Thirteen studies used an established theoretical framework to guide the development of data collection methods or data synthesis. Table 1 shows the characteristics of the included studies.

**Table 1:** Characteristics of included studies

**Lead author; year; country**	**Theoretical approach / framework**	**Study aim**	**Study design; Data collection method**	**Type of health professional**	**Practice setting**	**Participants**	**Barriers/ Enablers**	**TDF Domains** (number of barriers assessed in TDF domain)
**Smoking**								
Abatemarco [51] (2007) United States of America	Not stated	Identify the individual and environmental factors that enable or inhibit midwives’ ability to provide tobacco dependence treatment	Cross sectional study; Survey	Midwives	Hospital/ antenatal clinic; continuity model; 'Solo', 'Multi-specialty', 'Federal or state agency', 'Other'	193	Barriers	• Skills (1) • Optimism (1) • Beliefs about consequences (2) • Enviro, context and resources (4)
Bar-Zeev [24] (2017) Australia	Theoretical Domains Framework	Examine: (1) Self-reported provision of Smoking cessation care (SCC) to pregnant women by GPs and Obstetricians in Australia; (2) Barriers and enablers to SCC and (3) Associations between physician groups, knowledge, attitudes and performance of SCC	Cross sectional study; Survey	Mixed/unspecified medical practitioners	Primary care	378	Barriers and Enablers	• Optimism (1) • Beliefs about consequences (1) • Enviro, context and resources (2) • Social influence (1)
Bar-Zeev [52] (2019) Australia	Theoretical Domains Framework	Describe GPs' individual experiences with providing smoking cessation care to pregnant women who smoke and what would facilitate them to overcome known barriers	Qualitative; 1–1 interview	General Practitioners	Primary care	19	Barriers and Enablers	• Knowledge (2) • Skills (2) • Optimism (2) • Enviro, context and resources (2)
Beenstock [53] (2012) United Kingdom	Theoretical Domains Framework	Investigate perceived implementation difficulties of midwives in providing smoking-cessation advice to pregnant women who smoke, as specified in the NICE guidance	Cross sectional study; Survey	Midwives	Primary care; Hospital / antenatal clinic; High risk pregnancy	364	Barriers and Enablers	• Knowledge (2) • Skills (2) • Social/ professional role and ID (1) • Beliefs about capability (1) • Beliefs about consequences (2) • Goals (1) • Memory, attention, and decision processes (2) • Enviro, context and resources (1) • Social influence (1) • Emotion (2) • Behavioural regulation (1)
Coleman-Cowger [54] (2014) United States of America	Not stated	To assess current obstetrician-gynaecologist (ob-gyn) practice patterns related to the management of and barriers to smoking cessation during pregnancy and postpartum	Cross sectional study; Survey	Obstetricians	Hospital/ antenatal clinic; continuity model; Community health centre; 'solo/2-person practice', 'med school/uni', 'other'	470	Barriers	• Optimism (1) • Enviro, context and resources (2)
Colomar [55] (2015) Argentina and Uruguay	Not stated	Improve the understanding of systemic and individual factors influencing the implementation of the 5A's for smoking in prenatal care settings among prenatal clinic directors and providers in Argentina and Uruguay	Qualitative; 1–1 interview; Group interview	Multidisciplinary group: Midwives, Obstetricians, Clinical Directors, Nurses	Hospital / antenatal clinic	52	Barriers and Enablers	• Knowledge (3) • Skills (1) • Beliefs about capability (1) • Optimism (1) • Beliefs about consequences (1) • Enviro, context and resources (7)
DeWilde [56] (2015) Belgium	Not stated	Explore knowledge, beliefs and practice among midwives and gynaecologists concerning smoking cessation several years after the implementation of a smoking cessation policy for pregnant women and their partners	Qualitative; 1–1 interview	Multidisciplinary group: Midwives, Gynaecologists	Not stated	17	Barriers	• Skills (1) • Social/ professional role and ID (1) • Optimism (1) • Beliefs about consequences (2) • Enviro, context and resources (1)
Everett [57] (2005) South Africa	Not stated	Investigate current smoking cessation practices and attitudes of doctors working in public antenatal services, as well as their perceived barriers to addressing the issue in the context of routine care	Qualitative; 1–1 interview	Mixed/unspecified medical practitioners	Hospital / antenatal clinic	15	Barriers and Enablers	• Skills (3) • Optimism (3) • Beliefs about consequences (6) • Goals (1) • Enviro, context and resources (6)
Gould [58] (2020) Australia	Not stated	Assess medical practitioners’ agreement on what system-based changes (e.g., training, NRT access) could help improve their management of smoking in pregnancy	Cross sectional study; Survey	Mixed/unspecified medical practitioners	Primary care; ‘Obstetric care’	377	Enablers	• Skills (1) • Enviro, context and resources (3)
Grimley [59] (2001) United States of America	Transtheoretical Model of Behaviour Change	Determine the level of adherence to the clinical guidelines for smoking cessation among OB-GYN physicians within Alabama	Cross sectional study; Survey	Obstetricians	Group practice; Private practice; ‘other’	130	Barriers and Enablers	• Optimism (3) • Enviro, context and resources (1)
Hartmann [60] (2007) United States of America	Not stated	Assess the relationship between best practice and current intervention resources, prior training in smoking cessation intervention, and barriers to providing intervention	Cross sectional study; Survey	Multidisciplinary group: Midwives, Obstetricians, GPs, Nurse practitioners, Physician assistants	Not setting specific. The most common practice setting was groups of two to 10 providers (62%), primarily in single-specialty groups	549	Barriers	• Optimism (1) • Enviro, context and resources (1) • Social influence (1)
Herberts [61] (2012) England	Not stated	Juxtapose midwives’ perceptions of stop-smoking advice and service referral with pregnant smoker’s perceptions of the same stop-smoking service	Qualitative—Grounded theory; Social constructionist 1–1 interview; Group interview	Midwives	Hospital / antenatal clinic, ‘acute trusts’	15	Barriers and Enablers	• Knowledge (1) • Enviro, context and resources (1) • Social influence (2)
Hopman [62] (2019) Netherlands	Not stated	Assess the provision of quit-smoking counselling by midwives and clarify the nature and extent of any related barriers in Dutch midwifery settings	Cross sectional study; Survey	Midwives	Primary care; Hospital / antenatal clinic; ‘own practice’	135	Barriers	• Knowledge (2) • Skills (1) • Optimism (1) • Enviro, context and resources (5)
Jordan [63] (2006) United States of America	Not stated	Assess the degree to which obstetrician/ gynaecologists in the state of Ohio use best practices of smoking cessation with their pregnant patients who smoke	Cross sectional study; Survey	Obstetricians	Hospital / antenatal clinic; Private practice; ‘clinic’	125	Barriers	• Knowledge (1) • Enviro, context and resources (1)
Kalamkarian [64] (2022) Australia	Not stated	Understand midwives’ perspectives on current practices and their perceived barriers and facilitators to delivery of smoking cessation care	Qualitative; Group interview	Midwives	Hospital/ antenatal clinic; continuity model	53	Barriers and Enablers	• Social/ professional role and ID (1) • Optimism (4) • Beliefs about consequences (2) • Enviro, context and resources (2)
Longman [65] (2018) Australia	Theoretical Domains Framework	Explore the enablers and barriers to implementation of the antenatal smoking cessation guidelines among public health clinicians providing antenatal care	Qualitative; 1–1 interview	Multidisciplinary group: Midwives, Obstetricians, Maternity Service Managers	Hospital / antenatal clinic; continuity model; Aboriginal maternal and infant health service, Midwifery-led clinic, GP Shared Care, Obstetric Clinic	27	Barriers and Enablers	• Knowledge (1) • Skills (3) • Social/ professional role and ID (1) • Beliefs about consequences (2) • Enviro, context and resources (3) • Social influence (1)
O'Connell [66] (2014) United Kingdom	Not stated	Investigate midwives’ experiences of using carbon monoxide monitoring for smoking cessation in pregnancy as part of routine antenatal care	Qualitative—Descriptive phenomenology; 1–1 interview	Midwives	Not setting specific. Most midwives interviewed based in Greater or Central London	10	Barriers and Enabler	• Skills (2) • Social/ professional role and ID (1) • Optimism (1) • Reinforcement (1) • Enviro, context and resources (1) • Social influence (1)
Passey [67] (2020) Australia	Theoretical Domains Framework	Examine the association between midwives self-reported implementation of the 5As and reported barriers and enablers to their implementation	Cross sectional study; Survey	Midwives	Hospital/ antenatal clinic; continuity model; 'Midwife and GP Shared Care', 'Obstetric-led', 'Team midwifery', 'Aboriginal Maternal and Infant Health Service', 'Publicly funded home birth'	150	Barrier and Enablers	• Knowledge (4) • Skills (1) • Social/ professional role and ID (2) • Beliefs about capability (2) • Optimism (2) • Beliefs about consequences (6) • Intentions (3) • Goals (2) • Memory, attention, and decision processes (1) • Enviro, context and resources (3) • Emotion (2)
Price [68] (2006) United States of America	Not stated	Describe Ohio obstetricians/ gynaecologists’ perceptions and use of NRT	Cross sectional study; Survey	Obstetricians	Hospital/ antenatal clinic; Private practice; ‘HMO’ and ‘Academia’	154	Barriers	• Optimism (1)
Price [69] (2006) United States of America	Stages of change	Assess nurse-midwives in Ohio regarding their smoking cessation perceptions and practices for pregnant women who smoke	Cross sectional study; Survey	Midwives	Not stated	194	Barriers	• Knowledge (1) • Enviro, context and resources (1)
Reeks [70] (2020) Australia	Theoretical Domains Framework	Identify and understand the facilitators and barriers to providing smoking cessation support in antenatal care perceived by GPs	Qualitative; 1–1 interview	General Practitioners	Primary care	15	Barriers and Enablers	• Knowledge (2) • Beliefs about capability (1) • Beliefs about consequences (1) • Enviro, context and resources (1) • Social influence (2) • Emotion (1)
Thyrian [71] (2006) Germany	Not stated	Investigate the attitudes of midwives to counselling women about their smoking behaviour during pregnancy and postpartum	Cross sectional study; Survey	Midwives	Not stated	146	Barriers	• Optimism (1) • Enviro, context and resources (1) • Social influence (1) • Emotion (1)
**Alcohol**								
Anderson [72] (2010) United States of America	Not stated	Assess whether ob-gyns knowledge, practice, and attitudes have evolved during the past decade; estimate awareness and use of recently published tools; identify barriers for screening and intervention	Cross sectional study; Survey	Obstetricians	Hospital/ antenatal clinic; Community health centre; Private practice; 'group practice’, ‘solo practice’, ‘medical school or university’,‘other’	377	Barriers	• Beliefs about consequences (1) • Enviro, context and resources (1)
Chiodo [73] (2019) United States of America	Not stated	Describe midwives’ knowledge and attitudes toward the prevalence and risks of drinking alcohol during pregnancy, their practice in using clinical screening tools and perceived barriers to alcohol screening and intervention	Cross sectional study; Survey	Midwives	Hospital / antenatal clinic; continuity model; Community health centre; 'Solo/2 person', 'med school', 'birth centre' and 'student'	578	Barriers	• Skills (1) • Beliefs about consequences (2) • Enviro, context and resources (2)
Doherty [74] (2020) Australia	Theoretical Domains Framework	Assess antenatal clinician and manager barriers to the implementation of guidelines for addressing maternal alcohol consumption in antenatal services	Cross sectional study; Survey	Multidisciplinary group: Midwives, Obstetricians, Aboriginal Health Workers	Hospital / antenatal clinic	33	Barriers	• Skills (1) • Beliefs about capability (1) • Beliefs about consequences (1) • Enviro, context and resources (1) • Social influence (1) • Emotion (1) • Behavioural regulation (1)
Doi [75] (2014) United Kingdom	Not stated	Explore how midwives’ skills, knowledge and attitudes to alcohol consumption in pregnancy influence their practice; describe midwives’ practice in relation to screening and delivering of ABIs and identify barriers and facilitators	Qualitative; 1–1 interview	Midwives	Hospital / antenatal clinic	21	Barriers	• Skills (1) • Beliefs about consequences (2) • Enviro, context and resources (1)
Holmqvist [76] (2010) Sweden	Not stated	Evaluate how much education midwives in Sweden have undertaken to help them assess alcohol intake during pregnancy, and what tools they use to identify women who may be at risk of drinking during pregnancy	Cross sectional study; Survey	Midwives	Public maternity health-care centres	974	Enablers	• Skills (1) • Enviro, context and resources (4) • Social influence (1)
Olusanya [77] (2023) United States of America	Not stated	Describe midwives' knowledge, attitude and intent to screen for prenatal alcohol use and perceived barriers to communicating alcohol-related information	Cross sectional study; Survey	Midwives	Not stated	61	Barriers	• Knowledge (1) • Skills (1) • Beliefs about consequences (1) • Enviro, context and resources (3)
Scholin [78] (2021) United Kingdom	Theoretical Domains Framework	Explore midwives' views on implementation of the 2016 Chief Medical Officers' alcohol guidelines in antenatal care in the UK	Qualitative; 1–1 interview	Midwives	Hospital/ antenatal clinic; Community health centre; ‘Research or academic’	22	Barriers and Enablers	• Knowledge (2) • Social/ professional role and ID (1) • Beliefs about consequences (1) • Enviro, context and resources (2) • Social influence (2)
Smith [79] (2021) United Kingdom	Theoretical Domains Framework	Determine midwives’ knowledge of the CMO 2016 Guidelines and identify potential barriers and enablers of practice behaviour regarding asking and advising pregnant women about alcohol consumption	Cross sectional study; Survey	Midwives	Hospital/ antenatal clinic; ‘community or integrated team’, ‘Rotational’	842	Barriers	• Beliefs about capability (1) • Beliefs about consequences (2) • Social influence (2)
Wangberg [80] (2015) Norway	Not stated	Assess the current screening for and brief intervention on alcohol use in pregnancy among midwives in Norway, as well as perceived barriers for such practice	Cross sectional study; Survey	Midwives	Not specified	103	Barriers	• Knowledge (1) • Enviro, context and resources (3)
**Nutrition / Physical activity (PA) / Gestational weight gain (GWG) /**								
Asefa [81] (2020) Ethiopia	Not stated	Explore obstetricians’ and midwives’ views and practices related to GWG and postpartum weight management in health centre and hospital settings in Ethiopia	Qualitative; 1–1 interview	Multidisciplinary group: Midwives, Obstetricians	Hospital/ antenatal clinic; Community health centre	21	Barriers	• Knowledge (1) • Beliefs about capability (1) • Beliefs about consequences (3) • Enviro, context and resources (1)
Beulen [82] (2021) Netherlands	Not stated	Explore midwives’ perceptions of current and preferred nutrition communication practices in antenatal care, and identify what is needed to achieve their preferred practices	Qualitative- Appreciative inquiry; 1–1 interview	Midwives	Primary care; Hospital / antenatal clinic	20	Barriers and Enablers	• Social/ professional role and ID (1) • Beliefs about consequences (2) • Enviro, context and resources (3)
Chang [83] (2013) United States of America	Not stated	Understand the perceptions, approach, and challenges regarding management of GWG	Qualitative; 1–1 interview	Multidisciplinary group: Midwives, Obstetricians, Family Physicians	Community health centre; ‘Academic medical centre’	10	Barriers	• Knowledge (1) • Optimism (2) • Beliefs about consequences (3) • Enviro, context and resources (1)
Cheyney [84] (2010) United States of America	Not stated	Identify and examine how literature on pregnancy nutrition is incorporated into practice by obstetricians and midwives	Qualitative—Modified grounded theory; 1–1 interview; Observation of prenatal visits	Multidisciplinary group: Midwives, Obstetricians	Hospital/ antenatal clinic; Community health centre; ‘Independent freestanding birth centres’	24	Barriers	• Skills (1) • Social/ professional role and ID (1) • Enviro, context and resources (2)
Christenson [85] (2018) Sweden	Not stated	Explore how midwives approach communication about GWG recommendations with women and identify communication barriers and facilitators	Qualitative; 1–1 interview	Midwives	Hospital / antenatal clinic	17	Barriers	• Knowledge (1) • Skills (1) • Social/ professional role and ID (1) • Beliefs about consequences (3) • Enviro, context and resources (3)
DiStefano [86] (2021) Canada	Not stated	Investigate perceptions around GWG, nutrition, and PA among family practice obstetrics patients and providers. Highlight barriers to healthy GWG management	Qualitative; Group interview	Mixed/unspecified medical practitioners	Primary care; Community health centre	8	Barriers	• Optimism (1) • Beliefs about consequences (2) • Enviro, context and resources (1)
DeVivo [87] (2019) England	Not stated	Identify midwives perceived barriers to providing effective PA advice and guidance to pregnant women	Qualitative; 1–1 interview (component of a multiphase mixed methods study)	Midwives	Hospital / antenatal clinic	10	Barriers and Enablers	• Knowledge (3) • Skills (1) • Beliefs about consequences (2) • Enviro, context and resources (2)
Fieldwick [88] (2014) New Zealand	Not stated	Investigate the knowledge and practice of midwives providing lead maternity care regarding GWG	Qualitative; 1–1 interview; Group interview	Midwives	Hospital / antenatal clinic	12	Barriers and Enablers	• Beliefs about capability (1) • Optimism (4) • Beliefs about consequences (2) • Enviro, context and resources (7)
Fieldwick [89] (2019) New Zealand	Not stated	Explore the knowledge and practice of GPs with regards to GWG management and identify the current level of involvement GPs have in early pregnancy care	Cross sectional study; Survey	General Practitioners	Primary care	124	Barriers	• Skills (1) • Enviro, context and resources (3)
Hasted [90] (2016) Australia	Theoretical Domains Framework	Investigate barriers and enablers to routine weighing of women during pregnancy	Qualitative; Group interview	Multidisciplinary group: Midwives, Obstetricians	Hospital / antenatal clinic	42	Barriers and Enablers	• Skills (1) • Beliefs about capability (1) • Beliefs about consequences (4) • Enviro, context and resources (3)
Lindqvist [91] (2014) Sweden	Not stated	Explore how Swedish midwives experience the counselling of pregnant women on PA, specifically focusing on facilitators and barriers during pregnancy	Qualitative; Group interview	Midwives	Antenatal clinic. ‘Swedish ANC centres’	41	Barriers and Enablers	• Skills (3) • Beliefs about capability (2) • Optimism (2) • Enviro, context and resources (1) • Social influence (1)
Morris [92] (2017) Canada	Not stated	To understand current GWG counselling practices of healthcare providers, and the relationships between practices, knowledge and attitudes	Qualitative; 1–1 interview	Multidisciplinary group: general practitioners, nurses, midwives, obstetricians	Primary care	23	Barriers	• Knowledge (2) • Beliefs about consequences (2) • Enviro, context and resources (2)
Murray-Davis [93] (2020) Canada	Not stated	To understand the counselling among antenatal HCPs in Ontario and what factors act as barriers and enablers to the provision of counselling about GWG	Qualitative—Grounded theory; 1–1 interview	Multidisciplinary group: Midwives, Obstetricians, General Practitioners	Not specified	18	Barriers	• Skills (1) • Beliefs about consequences (2) • Enviro, context and resources (3)
Stotland [94] (2010) United States of America	Not stated	Explore and describe prenatal care providers’ knowledge, attitudes, and practices regarding GWG, nutrition, and PA counselling. Identify and characterize barriers to weight gain counselling	Qualitative; Group interview	Multidisciplinary group: Midwives, Obstetricians, Nurse Practitioners	Hospital/ antenatal clinic; Private practice; ‘academic health centres’, ‘health maintenance organisation’	52	Barriers	• Skills (1) • Beliefs about capability (1) • Optimism (1) • Beliefs about consequences (1) • Memory, attention, and decision processes (1)
Timmerman [95] (2017) United States of America	Not stated	Examined OBs' practices for managing GWG along with perceived barriers	Cross sectional study; Survey	Obstetricians	Hospital/ antenatal clinic; Private practice	63	Barriers	• Beliefs about consequences (2) • Enviro, context and resources (3)
Van der Pligt [96] (2011) Australia	Not stated	Assess GP’s perspectives regarding the management and assessment of GWG and to understand how GPs can be best supported to provide healthy GWG advice	Qualitative—Descriptive; 1–1 interview	General Practitioners	Primary care	28	Barriers and Enablers	• Social/ professional role and ID (1) • Beliefs about consequences (2) • Enviro, context and resources (6)
Walker [97] (2019) Australia	Theoretical Domains Framework and COM-B	Explore the perceptions and experiences of GPs in Australia in relation to implementing GWG recommendations in GP-led antenatal care	Qualitative- Exploratory; 1–1 interview	General Practitioners	Primary care	20	Barriers and Enablers	• Knowledge (1) • Social/ professional role and ID (1) • Optimism (3) • Beliefs about consequences (2) • Enviro, context and resources (3) • Social influence (1)
Willcox [98] (2012) Australia	Not stated	Explore midwives' views, attitudes and approaches to the assessment, management and promotion of healthy GWG and to investigate their views on optimal interventions	Qualitative- Descriptive; 1–1 interview	Midwives	Hospital/ antenatal clinic; Group practice (continuity model); Community health centre; 'GP shared care’	14	Barriers and Enablers	• Social/ professional role and ID (1) • Beliefs about capability (1) • Beliefs about consequences (3) • Enviro, context and resources (3)

### Study quality assessment

The quality assessment of included studies is provided in Additional file 4. Each study included clear research questions and reported on data that addressed those questions, passing the two screening questions. Of the 27 qualitative studies, most used adequate data collection methods to address the research question, included an interpretation of results supported by data, and provided a clear link between data source/s, collection, analysis, and interpretation (59%-74% met the criteria). The most frequently met criteria for qualitative studies was ‘Are the findings adequately derived from the data?’ (74%), reflecting high quality reporting of analytic procedures. However, qualitative studies were less likely to demonstrate that the chosen qualitative approach was appropriate to answer the research question, with only one-third meeting this criterion (33%) and most being rated as ‘can’t tell’ (55%), reflecting poor articulation in methodology. Of the 22 quantitative descriptive studies, most met the criteria related to using an appropriate sampling strategy and statistical analysis to address the research question (86%-95% met criteria). However, they did not demonstrate that participant samples were representative of the target population, the use of appropriate measures, and low risk of nonresponse bias (62%-76% failed to meet criteria). Overall, the methodological quality of qualitative studies was assessed as higher than that of quantitative studies. The number of criteria met by the quantitative studies ranged from 0–5, with a mode 4/5, median 4 and mean 3. The number of criteria met by the quantitative studies ranged from 1–4, with mode 2/5, median 2 and mean 3.

### Results of individual studies

**Fig. 2:**
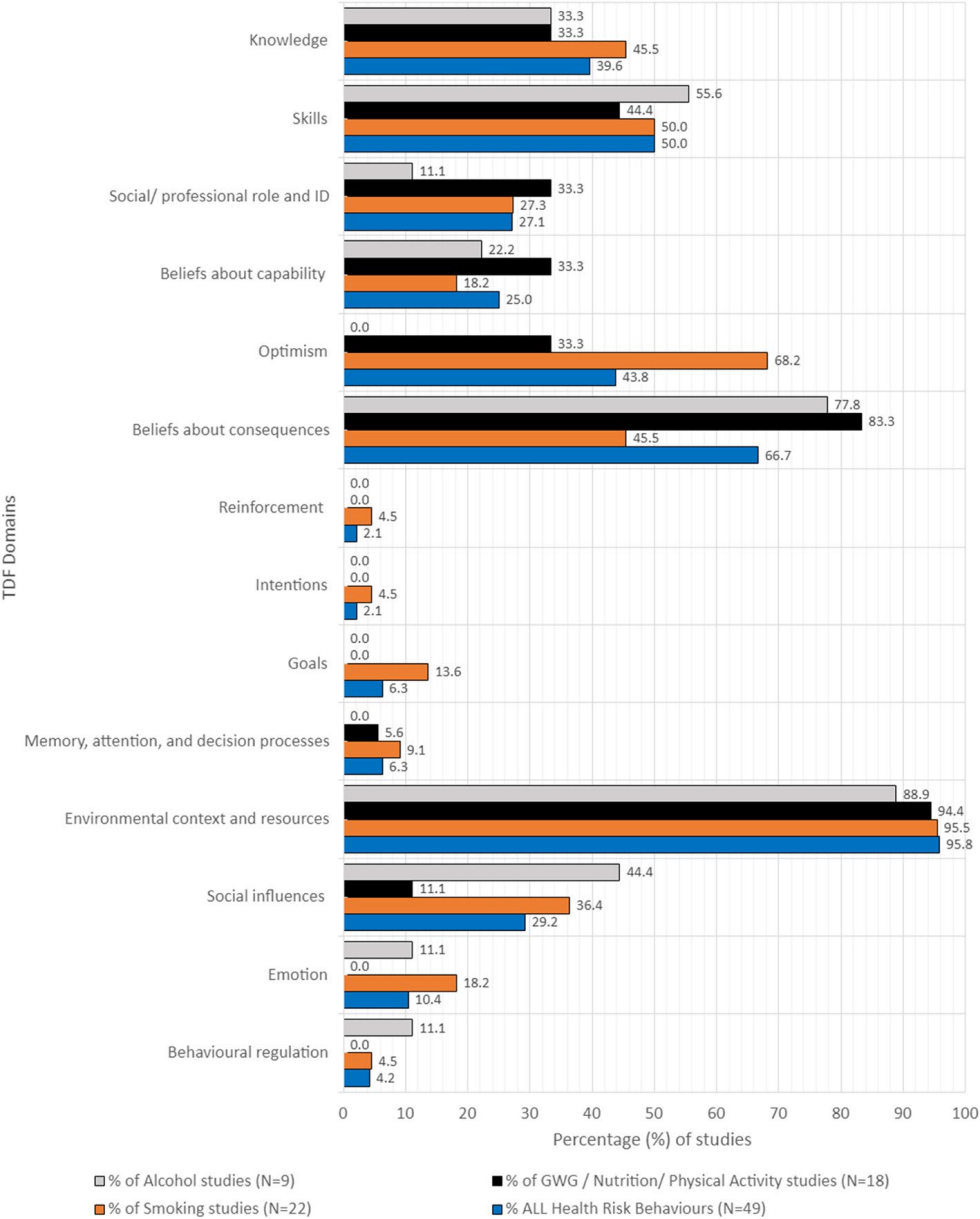
Barriers and enablers by SNAP-W health risk behaviour all antenatal professionals

A total of 352 barriers and enablers were extracted from the 49 included studies. The number of barriers and enablers extracted from individual studies ranged from one [68] to 28 [67] with a median of six barriers/enablers per study. Twenty-six studies reported barriers only, twenty reported barriers and enablers and two studies reported enablers only. The number of TDF domains that the barriers/enablers from individual studies covered ranged from one domain [68] to 11 [53, 67] with a median four domains reported in each study. Additional files 5, 6, 7. provide details of the barriers and enablers as reported in the individual studies. Barriers/enablers to care addressing SNAP-W health risks were coded to all 14 TDF domains Fig. 2 presents the frequency of barriers and enablers within each TDF domain as a percentage of the included studies. Determinants are reported for each SNAP-W risk behviour and across all SNAP-W health risk behaviours.

### Synthesis of results

#### Smoking related antenatal care barriers and enablers

Twenty-two included studies, published between 2001 and 2022, examined the barriers and enablers to smoking cessation care during pregnancy. Barriers and enablers were reported across all 14 of the TDF domains. The most common TDF domains were ‘environmental context and resources,’ which was reported in 95.5% (*n* = 21) of studies [24, 51, 53, 55–67, 69–71]; followed by ‘optimism’ (68.2%; *n* = 15), which often reflected barriers related to ‘pessimism’ [24, 51, 52, 54–57, 59, 60, 62, 64, 66–68, 71]), ‘skills’ (50%; *n* = 11) [51–53, 55–58, 62, 65–67], ‘knowledge’ (45.5%; *n* = 10) [52, 53, 55, 61–63, 65, 67, 69, 70], and ‘beliefs about consequences’ [51, 53, 55–57, 64, 65] (45.5%; *n* = 10).

‘Environmental context and resources’ and ‘optimism’ were consistently the top domains identified across all antenatal care provider groups, including midwives (*n* = 9) [51, 53, 61, 62, 64, 66, 67, 69, 71], multidisciplinary groups (*n* = 4) [55, 56, 60, 65], obstetricians/gynaecologists (*n* = 4) [54, 59, 63, 68], mixed medical officer groups (*n* = 3) [24, 57, 58], and general practitioners (*n* = 1) [52].

Within the ‘environmental, context and resources’ domain, sufficient time was perceived as an enabler [67] and insufficient time as a barrier to providing smoking cessation care [24, 51, 54–57, 60–67, 69–71]. Reimbursement, in the form of billable Medicare item numbers for cessation counselling was an enabler [58] and the lack of reimbursement/ remuneration for consultation was a barrier [55, 62]. Other financial factors included cost and access to medication (e.g., Nicotine Replacement Therapy) [51, 52, 58]. Seven studies identified access to physical resources, including written material and attractive visual items, as factors that influenced delivery of care [52, 55, 57, 59, 62, 67].

Organisational contextual factors included the use of an electronic medical record [65], structures and processes that prioritised smoking cessation, including guidelines and continuity of care models [55, 64, 70] and access to referral supports [51, 54]. Barriers were reported that highlighted the impact of the broader system and organisational context, including levels of stress, working conditions and acute shortage of midwives in public sector antenatal services [57].

Within the ‘optimism’ domain, barriers and enablers related to professional’s confidence that things would happen for the best or desired goals would be attained. Factors reported in this domain were labelled optimism [24, 67], pessimism [57], scepticism or futility [64]. More often the barrier was expressed as a lack of confidence in achieving desired outcomes [24, 51, 54–57, 59, 60, 62, 64, 66, 68, 71]. At times these perceptions caused reluctance to provide smoking cessation support [56].

### Alcohol consumption related antenatal care barriers and enablers

Nine studies, published between 2010 and 2023, examined the barriers and enablers to addressing alcohol consumption within routine antenatal care [72–80]. Barriers and enablers were coded to nine of the TDF domains. The most common domain was ‘environmental context and resources’ (88.9%; *n* = 8) [72–78, 80], followed by ‘beliefs about consequences’ (77.8%; *n* = 7) [72–75, 78], then ‘skill’ (55.6%; *n* = 5) [73–77] and ‘social influence’ (44.4%; *n* = 4) [74, 76, 79, 99].

Seven of the nine studies reported the perspectives of midwives [73, 75–80], resulting in midwife’s perspectives heavily influencing the identified domains. Of the remaining two studies, Doherty, Kingsland [74] reported perspectives of a multidisciplinary sample and Anderson, Dang [72] sampled obstetricians and gynaecologists.

Insufficient time was the most common barrier coded to the ‘environmental context and resources’ domain [72, 73, 75, 77, 78, 80]. Other barriers included lack of organisational support [80] and poor resources [73, 77]. Enablers coded to the ‘environmental context and resources’ domain related to improved access to guidelines [76], time for extra consultations with a midwife [76] and the completion of a validated screening tool prior to appointment attendance [78].

Barriers coded to the ‘beliefs about consequences’ domain [72–75, 77, 78], included patient denial/ resistance to treatment [72, 73], patient sensitivity [73, 77], overload of information at the initial antenatal appointment [75], and competing workload priorities [75], where alcohol was reported as a low priority because of the lack of perceived impact of alcohol consumption on fetal outcomes [75].

A trusting therapeutic relationship between midwives and their patients was reported as an enabler [78]. Clear, effective, and compassionate communication required non-confrontational discussions to ensuring that pregnant people feel comfortable disclosing alcohol use without being stigmatised [78].

The domain of **‘**social influence’ was coded as a determinant to care related to alcohol consumption more often than for care related to other health risk behaviours (44% alcohol; 11% nutrition/ physical activity/ gestational weight gain; and 36% smoking) [74, 76, 79, 99]. Different advice about alcohol consumption in pregnancy provided by health professionals was a barrier [78].

### Nutrition/ physical activity/ gestational weight gain barriers and enablers

There were 18 studies, published between 2010 and 2021, that reported barriers and enablers to antenatal care provision related to nutrition/ physical activity/ gestational weight gain in pregnancy [81–84, 86–98, 100]. Barriers and enablers were coded to nine of the 14 TDF domains. The barriers were most frequently coded to the ‘environmental context and resources’ domain (94.4%; *n* = 17) [81–84, 86–93, 95–98, 100], followed by the ‘beliefs about consequences ‘ domain (83.3%; *n* = 15) [81–83, 86–88, 90, 92–98, 100] and the ‘skills’ domain (44.4%; *n* = 8) [84, 87, 89–91, 93, 94, 100].

Seven studies reported barriers and enablers reported by multidisciplinary participants [81, 83, 84, 90, 92–94], six by midwives [82, 87, 88, 91, 98, 100], and three by general practitioners [89, 96, 97]. Timmerman, Walker [95] reported perceived barriers of obstetricians and Di Stefano, Godard [86] reported the barriers of physicians who were not otherwise specified as specialist medical practitioners. Consistently, across all antenatal care provider groups, factors related to the ‘environmental context and resources’ and ‘beliefs about consequences’ were the most prominent domains to influence their practice related to nutrition/ physical activity/ gestational weight gain in pregnancy. Like care addressing alcohol consumption and smoking, barriers within the ‘environmental context and resources’ domain included insufficient time [81, 88, 93, 95–97, 100]. In contrast, more determinants coded to this domain related to resources and organisational context. Studies highlighted a complex interplay of resources need and organisational constraints [84], including access to referral services/ inter-professional collaboration or multidisciplinary support [82, 87, 88, 92, 95, 96, 98]; the cost of referral support [93, 95]; access to scales (for assessing weight) [88, 90]; difficulties with systems and documentation [90]; and availability of appropriate patient resources [82, 83, 87, 93, 95–98, 100], including unsuitable languages [86]. Organisational contextual barriers and enablers included appointment schedules and times [84, 91, 92, 97, 100], funding [88], availability of continuity models of care [84, 88, 90, 98], and inter-professional collaboration [82, 87, 90].

Many barriers coded to the ‘beliefs about consequences’ domain [81–83, 86–88, 90, 92–98, 100] were about the perceived sensitivity of weight as a topic [81–83, 86–88, 92–94, 98, 100], including concerns about weight stigma [90, 100] and fear of offending patients [87]. Other barriers in the ‘beliefs about consequences’ domain included care being considered a low priority [81–83, 92, 97, 98], and questioning the evidence for care provision [90].

### Reporting biases

Due to a lack of published protocols, it was not possible to determine the risk of reporting bias.

## Discussion

The findings of this review highlight some consistency in the barriers and enablers to antenatal care reported across the SNAP-W health risks, as well as some notable differences. Barriers and enablers within the TDF domain ‘environmental context and resources’ were identified in around 96% of studies across each of the SNAP-W health risks. While this is a broad domain, there was consistency across studies, in identifying time, access to and quality of resources, and organisational supports as key determinants. Beyond this domain, differences in determinants were apparent for different SNAP-W risk factors and antenatal professional groups. Notably, almost 70% of studies related to smoking cessation care reported barriers coded to the ‘optimism’ domain, over 80% of studies on care related to nutrition/ physical activity/ gestational weight gain reported barriers coded to the ‘beliefs about consequences’ domain, and almost 50% of studies of care related to alcohol consumption and 40% related to smoking cessation care reported barriers coded to ‘social influence’. The ‘environmental context and resource’ was the leading domain across all antenatal care provider groups.

The predominance of barriers associated with ‘environmental, context and resources’ was consistent with previous systematic reviews of antenatal care related to smoking [34] and alcohol [35], which reported barriers including organisational context [34], time constraints, and lack of clear protocol [35]. These findings confirm those of several other systematic reviews which have found that barriers and enablers related to ‘environmental context and resources’ are common determinants of guideline recommended care delivery across diverse clinical settings [36, 101, 102]. The review findings demonstrate the importance of the systems, organisational structures and protocols within which health service staff operate in influencing the care that individual healthcare providers deliver, and that such external determinants are generally more influential than internal determinants related to the individual healthcare provider’s motivation and capability.

The specific determinants found for individual health risks were also supported somewhat by previous reviews. For instance, we found that for care related to nutrition/ physical activity/ gestational weight gain ‘beliefs about consequences’ was the second most frequently reported domain, which was also found by Heslehurst, Newham [36] who conducted a mixed methods systematic review to identify determinants related to maternal obesity and weight management [36]. These findings are consistent with a large body of growing evidence regarding weight stigma [103] and may be influencing beliefs and behaviours of clinicians regarding care associated with gestational weight gain. Similarly, we found ‘beliefs about consequences’ to be an important domain in relation to care related to alcohol consumption. This was consistent with a systematic review of barriers to screening for alcohol or other drugs during pregnancy conducted by Oni, Buultjens [35]. The review reported perceived barriers related to concerns about damaging the therapeutic relationship and causing anxiety or guilt by asking about alcohol consumption and perceived inconclusive evidence regarding alcohol consumption during pregnancy. In relation to smoking related care, consistent with our findings, a review by Flemming, Graham [34] reported scepticism and pessimism as a barrier, but this was integrated across their major themes related to the professional role and the organisational context.

We found that there were some differences in determinants to antenatal SNAP-W care based on health professional groups. While all groups reported ‘environmental context and resources’ as influential, 'beliefs about consequences' was the second most common domain for midwives, multidisciplinary practitioners and general practitioners 'Optimism', largely represented by pessimistic views, was the second most common domain for obstetricians,gynaecologists, and other mixed medical samples. While we found no previous reviews on SNAP-W antenatal care that reported on determinants by profession, these findings are consistent with broad literature regarding professional differences in determinants to care [104]. Such differences may be reflective of fundamental core differences in the disciplines, training and practices of midwives and nursing professions and medical professions [105, 106].

### Limitations of the evidence

The overall quality of the included studies varied. More qualitative studies than quantitative studies met all the respective quality appraisal criteria. Most quantitative studies were at risk of non-response bias due to low response rates, suggesting that their findings might not be representative of antenatal care providers broadly. Included studies that did not use a theoretical framework are at risk of confirmation bias due to their reliance on the outcomes of previous research to inform their survey development or question guide or the simple selection of a specific domain of interest without justification [107]. It is also worth noting that older studies within the review may have limited utility in the current context considering changes to policy and evidence to support treatment, for example in relation to Nicotine Replacement Therapy [51], and Fetal Alcohol Syndrome [75], which now have an established evidence base and are embedded within smoking cessation and alcohol abstinence pregnancy care guidelines [20].

### Limitations of the review

This review has many strengths, including the use and synthesis of data from both quantitative and qualitative studies, the inclusion of studies examining both enablers and/or barriers for a more comprehensive assessment, and grounding the synthesis in a theoretical framework, the TDF. However, findings should be interpreted with consideration of its limitations. There is potential that the final study sample does not represent all relevant research. For example, the search was conducted in English only, which may have contributed to the small number of studies from low and middle-income countries (higher prevalence of non-English speaking). Without such representation, the external validity of the review findings may be restricted to high-income countries. Despite using a broad and comprehensive search and dual independent reviewers undertaking screening and selection, it is possible that studies reporting barriers/enablers as a part of larger studies and trials may have been excluded. The review team observed that qualitative studies were often less explicit in identifying barriers/enablers as part of their aims statement, which may have resulted in exclusion. It is possible that publication bias exists; noting that only one qualitative study was published before 2010 [57]. Finally, the variety of measures, and lack of consistency in approaches to elicit barriers and enablers required the review team to nominate an arbitrary cut point of 30% to identify priority barriers in the absence of any evidence to base a cut point on. It is possible that in applying this cut point, some minor barriers and enablers were excluded from the synthesis.

### Implications for future research, practice and policy

Implementation science-based approaches to supporting improvement to practices recommend implementation strategies be developed based on a theoretical understanding of barriers and enablers [27, 28]. However, only 13 of 49 included studies utilised an evidence-based theoretical framework to examine barriers and enablers [24, 52, 53, 59, 65, 67, 69, 70, 74, 78, 79, 90, 97] (typically the TDF). Without the use of a theoretical framework such as the TDF [37, 50] or Consolidated Framework for Implementation Research [108], there is risk that studies direct and isolate their enquiries to a subset of potential barriers that only cover limited domains and introduce confirmatory bias. Implementation strategies developed based on the barriers elicited through a biased approach can therefore be ineffective as they may not be designed to address true barriers to care. Future research into the determinants (barriers/enablers) of care delivery related to SNAP-W should utilise theoretical frameworks so that a comprehensive assessment of determinants can be undertaken and be used to support the development of effective implementation strategies [109, 110]. Strong representation of studies from high income countries, coupled with the heterogeneity of clinical settings within the included studies and the high proportion of barriers within the ‘environmental, context and resources’ domain support the need for future intervention development to explore determinants specific to the local context. Similarly, although out of the scope of this review, consumer, and local stakeholder engagement, including policy and practice partners is important to ensure that interventions and implementation strategies are appropriate to the implementation setting.

The findings of this review suggest that implementation strategies to improve antenatal SNAP-W care should fundamentally target barriers in the ‘environmental, context and resources’ domain. Strategies that [33, 111–113] may be effective include ‘changing the physical structure and equipment’, ‘restructuring the physical environment’ or ‘adding objects to the environment’ [113, 114] to address time barriers created, as described by Hasted, Stapleton [90] as not having “the stuff handy”. These strategies could also be applied to address barriers related to access to high quality and appropriate physical patient resources [52, 55, 57, 59, 62, 67, 73, 77, 82, 83, 87, 93, 95–98, 100], and access to scales to measure patient’s weight [88, 90], which may additionally act as a physcial ‘promtp or cue’ to weigh patients [113]. ‘Social support (practical)’, ‘restructuring of the social environment’ and again ‘adding objects to the environment’ [113] through the addition or availability of clear structured local processes, guidelines and policy to support practice [55, 57, 67, 76, 88, 90, 97], along with electronic medical records systems, with integrated validated health risk screening tools [65, 78] may be effective in improving care. The availability of models of care that provide continuity were also highlighted as an important factor to support SNAP-W care [64, 70, 84, 88]. ‘Restructuring the physical / social environment’ to make such models available would also align with longstanding strong evidence to support continuity models to improve many maternal outcomes, including reduced birthing intervention and increased satisfaction [115].

To make significant improvements to care delivery, researchers, service providers and policy makers need to consider important secondary domains when developing and implementing strategies to improve SNAP-W care. These secondary determinants differed by health risk and health professional discipline, including ‘optimism’ for smoking, obstetricians, gynaecologists, and other mixed medical samples; ‘beliefs about consequences’ for nutrition/ physical activity/ gestational weight gain and midwife, multidisciplinary and general practitioner samples; and ‘social influence’ for alcohol. Training and education strategies may be effective [33, 108–110] in addressing barriers in the ‘optimism’ and ‘beliefs about consequences’ domains if they include behaviour change techniques such as persuasive reinforcement about research evidence and the salience of preventive care during pregnancy [33, 113]. For example, highlighting preventive care as cost effective [116], highly acceptable interventions [117, 118] that improve health outcomes for pregnant people and their babies [119].

Within this review determinants related to time and cost were coded to the ‘environmental, context and resources’ domain. However, parallels can be drawn between determinants in the ‘environmental, context and resources’ domain and the ‘beliefs about consequences’ domain. For example, cost versus perceived benefit and lack of time from the perspective of prioritisation of preventive care (making time). As such, strategies including policy and funding models that ‘incentivise’ the delivery of preventive care may address barriers related to the cost of medication (e.g., Nicotine Replacement Therapy) [51, 52, 58], cost of referral and multidisciplinary support [82, 87, 88, 92, 93, 95, 96, 98], and lack of reimbursement for clinicians’ time providing smoking cessation counselling [55, 58, 62] may be effective.

## Conclusion

This review highlighted influential determinants of healthcare professionals’ behaviours in relation to the routine delivery of antenatal care addressing SNAP-W risk factors for pregnant people. Barriers and enablers within the TDF domain of ‘environmental context and resources’ were identified as the most influential. Implementation Strategies that modify the environment, such as restructuring appointments, improving resource provision and improving clinical support systems, are therefore essential if any significant differences are to be made to improve the delivery of recommended care to pregnant people related to SNAP-W risks. To further support improvement, there is also a need for implementation strategies tailored to notable differences in secondary determinants related to specific health risk behaviours and antenatal care provider groups. Testing the effectiveness of these theoretical strategies in implementation trials in multidisciplinary antenatal care settings in an essential next step in progressing the field and improving care delivery so that preventive antenatal health care for smoking, nutrition, alcohol, physical activity and gestational weight gain is provided routinely and the benefits of such care to pregnancy and newborn outcomes are realised.

## Supplementary Information

Supplementary Material 1.

Supplementary Material 2.

Supplementary Material 3.

Supplementary Material 4.

Supplementary Material 5.

Supplementary Material 6.

Supplementary Material 7.

## Data Availability

The datasets used and/or analysed during the current study are available from the corresponding author on reasonable request.
