# Supplementary material for: Barriers and enablers to addressing smoking, nutrition, alcohol consumption, physical activity and gestational weight gain (SNAP-W) as part of antenatal care: A mixed methods systematic review
Source: Implement Sci Commun. 2024 Oct 9;5:112. doi: 10.1186/s43058-024-00655-z (PMC11462853; doi:10.1186/s43058-024-00655-z)
Supplement: Supplementary file 2 — Supplementary Material 2. [file 43058_2024_655_MOESM2_ESM.pdf]

Additional File 2. Medline search strategy

Ovid MEDLINE(R) and Epub Ahead of Print, In-Process, In-Data-Review & Other Non-Indexed Citations and Daily <1946 to November 23, 2022>

1 pregnancy/ 967048

2 Maternal Behavior/ 12089

3 Pregnant Women/ 13237

4 Prenatal Care/ or antenatal.mp. 65988

5 1 or 2 or 3 or 4 988910

6 "Delivery of Health Care"/ 112122

7 Maternal health service/ 15947

8 matern* health service*.mp. 17012

9 Family Planning Services/ 26154

10 Midwifery/ 20884

11 Nurse Midwives/ 7485

12 Physician, Family/ or family Practice/ or General Practice/ or General Practitioners/ 99324

13 Obstetric Nursing/ 3063

14 "Obstetrics and Gynecology Department, Hospital"/ or Obstetrics/ 26803

15 ((Health or healthcare) adj2 (profession* or worker*)).mp. 199104

16 Health Services, Indigenous/ 3963

17 6 or 7 or 8 or 9 or 10 or 11 or 12 or 13 or 14 or 15 or 16 482221

18 Gestational Weight Gain/ or Weight Gain/ or Obesity/ or Body Mass Index/ 331272

19 (pregnancy weight gain or gestational weight gain).mp. 4454

20 18 or 19 332417

21 Fetal alcohol spectrum disorders/ or f?etal alcohol spectrum disorder*.mp. or FASD.mp. 5477

22 alcohol abstinence/ 885

23 alcohol*.mp. 478730

24 Drinking behavior/ or exp alcohol drinking/ or drinking/ or binge drinking/ 96728

25 21 or 22 or 23 or 24 498681

26 Smoking Reduction/ or Tobacco Smoking/ or Smoking Cessation Agents/ or Cigarette Smoking/ or Smoking Cessation/ or Smoking/ or Smoking Prevention/ 172700

27 (smo* adj2 (prevent* or reduc* or cessation or cease* or cigarette or tobacco)).mp. 131915

28 Tobacco products/ 7288

29 (nicotine adj1 replacement).mp. 3977

30 NRT.mp. 2531

31 "Tobacco Use Cessation"/ or Tobacco/ or "Tobacco Use"/ or "Tobacco Use Cessation Devices"/ 41001

32 26 or 27 or 28 or 29 or 30 or 31 249411

33 Diet, Healthy/ or Diet/ or food/ or "Diet, Food, and Nutrition"/ 219336

34 Energy intake.mp. or Energy Intake/ or dietary guideline.mp. or Nutrition Policy/ 65926

35 33 or 34 265697

36 exp Exercise/ 238274

37 Sedentary Behavior/ 13099

38 (sedentary adj2 (behavio* or lifestyle*)).mp. 21741

39 physical inactivit*.mp. 9995

40 Physical Fitness/ 29378

41 Motor Activity/ 99839

42 sitting time.mp. 1673

43 36 or 37 or 38 or 39 or 40 or 41 or 42 359956

44 20 or 25 or 32 or 35 or 43 1536243

45 "Attitude of Health Personnel"/ or exp Attitude to Health/ or Health Services Accessibility/ or exp "behavior and behavior mechanisms"/ or exp Professional-Patient Relations/ 3341571

46 ("enabler*" or "barrier*" or "facilitator*" or "perception*" or "opinion*" or "perspective*").m_titl. 335384

47 ((point adj1 view) or point-of-view).tw. 49611

48 45 or 46 or 47 3607569

49 exp "Delivery of Health Care"/ 1202450

50 Implementation Science/ or implementation.mp. 328501

51 organi?ational change*.mp. 3554

52 (system* adj2 change*).mp. 19529

53 quality improvement*.mp. 68083

54 Mass Screening/ 115063

55 Outcome Assessment, Health Care/ or Process Assessment, Health Care/ or Health Impact Assessment/ or "Outcome and Process Assessment, Health Care"/ or Patient Outcome Assessment/ or Nursing Assessment/ 149168

56 Health Education/63231

57 "Referral and Consultation"/ 74792

58 brief intervention.tw. or Motivational Interviewing/ or Psychotherapy, Brief/ 8887

59 5As.mp. 511

60 ((Care or practi?e*) adj (best or evidence* or recomm*)).tw. 7690

61 Health Behavior/ or Health Promotion/ or Public health/214378

62 49 or 50 or 51 or 52 or 53 or 54 or 55 or 56 or 57 or 58 or 59 or 60 or 61 1961075

63 5 and 17 and 44 and 48 and 62 941
