# Supplementary material for: Barriers and enablers to addressing smoking, nutrition, alcohol consumption, physical activity and gestational weight gain (SNAP-W) as part of antenatal care: A mixed methods systematic review
Source: Implement Sci Commun. 2024 Oct 9;5:112. doi: 10.1186/s43058-024-00655-z (PMC11462853; doi:10.1186/s43058-024-00655-z)
Supplement: Supplementary file 5 — Supplementary Material 5. [file 43058_2024_655_MOESM5_ESM.pdf]

Additional File 5. Barriers and enablers reported by study authors coded to TDF - SMOKING

TDF Domain (definition)	Barriers	Enablers
**1. Knowledge**<br>(An awareness of the existence of something)	• Subtheme: Needing clear and detailed information [52][1]<br>• Barriers for Quitline referral [52]<br>• Provider: Knowledge:<br>- Lack of information on the risks of smoking during pregnancy<br>- Perception that there is no problem in current practice [55]<br>• Midwives - knowledge/training (Barrier/ Enabler) [61]<br>• I have all the required information about nicotine replacement products and pharmacotherapeutics.* reverse scored - % barrier strongly disagree with this statement [62]<br>• I have a clear picture of referral options regarding quit-smoking counselling in the neighbourhood.* reverse scored strongly disagree reported [62]<br>• Not knowing where to send patients for treatment [63]<br>• Knowledge [65]<br>• Not knowing where to send the pregnant smoker for treatment [69]<br>• Professional attributes: Knowledge of the 5As [70]<br>• Professional attributes: Knowledge and confidence regarding NRT [70]	• Knowledge [53]<br>• Provider: Providing education on the benefits of smoking cessation would lead to willingness to change behaviour [55]<br>• I know how to provide smoking cessation support in antenatal care to help pregnant women quit [67]<br>• I have good knowledge of nicotine addiction and the barriers to quitting smoking [67]<br>• I am familiar with the guidelines for using the 5As for smoking cessation during antenatal care (Ask, Advise, Assess, Assist, Arrange Follow-up) [67]<br>• I have good knowledge of the harms of smoking in pregnancy [67]
**2. Skills**<br>(An ability or proficiency acquired through practice)	• Lack of training or experience in smoking cessation [51]<br>• Current practices were sub-optimal [52]<br>• Needing better communication skills [52]<br>• Clinics: Inadequate training to provide counselling [55]<br>• Advise and Assess - Lack of communication skills [56]<br>• Little understanding of how to motivate patients to change behaviour [57]<br>• Lack of smoking cessation counselling skills and educational aids [57]	• Skills [53]<br>• Doctors keen to improve communication with pregnant women about smoking [57]<br>• Doctors concerned to adopt more empathetic, patient-centred approach to smoking cessation counselling [57]<br>• Doctors expressed need for training in behavioural change counselling skills to use in variety of situations [57]

[1] To find the referenced study refer to the reference list in main article.

	• I have sufficient skills to discuss quitting smoking* reverse scored - % barrier strongly disagree with this statement [62]<br>• Skills [65]<br>• Training [65]<br>• Tick box exercise [66]<br>• Lack of training [66]	• Health professional training [58]<br>• I have good skills in assisting pregnant women with strategies to quit smoking [67]<br>•
**3. Social/professional role and identity** (A coherent set of behaviours and displayed personal qualities of an individual in a social or work setting)	• Advise - Giving advice is not my job [56]<br>• Expanding roles [66]	• Professional role/identity [53]<br>• Opportunities to enable midwives' ability to provide smoking cessation care - Defining responsibilities and the role of the midwife [64]<br>• Professional role and identity [65]<br>• Helping women quit smoking makes me feel proud of my role [67]<br>• Providing smoking cessation support for pregnant women is an important part of my role [67]
**4. Beliefs about capabilities** (Acceptance of the truth, reality or validity about an ability, talent or facility that a person can put to constructive use)	• Provider: Attitude: Lack of self-efficacy to address problem [55]	• Beliefs about capabilities [53]<br>• I am confident providing smoking cessation assistance to pregnant women [67]<br>• I am confident assessing women's smoking status [67]<br>• Professional attributes: Beliefs about capabilities [70]
**5. Optimism** (The confidence that things will happen for the best or that desired goals will be attained)	• Lack of patient interest [51]<br>• Mixed feelings regarding managing smoking during pregnancy (Barrier/ Enabler) [52]<br>• Barriers for NRT prescription [52]<br>• Expected patient denial or resistance to treatment [54]<br>• Provider: Beliefs: Poor outcome expectancies for smoking cessation strategies [55]	• I feel optimistic that providing smoking cessation support helps women quit smoking [67]<br>• Providing smoking cessation support to women is not worth it given the small level of success – reverse scored [67]

	• Advise - Disappointment: no effect of advice, no influence on the woman [56]<br>• Pessimism among health care providers that they can influence patient behaviour [57]<br>• Negative attitude of pregnant women towards quitting [57]<br>• Apparent lack of concern about smoking in pregnancy among women [57]<br>• Patients may not be motivated to quit; therefore, advising them is a waste of time [59]<br>• If a patient is still smoking at the end of her first trimester, then she probably will not quit at all [59]<br>• A patient may have some major stresses that smoking may help to relieve [59]<br>• Limited effectiveness of smoking intervention [60]<br>• I find it difficult to discuss quitting smoking with certain clients since they simply cannot be motivated [62]<br>• Scepticism and doubt in SCC provision [64]<br>- Sense of futility about affecting changes in smoking behaviour<br>- The role of social normalisation.<br>- Sense of futility about affecting changes in smoking behaviour - Pregnant women's denial of smoking risks Scepticism and doubt in<br>- Not able to trust pregnant women's disclosures of smoking behaviours<br>- The stigma and silence of smoking<br>- Not able to trust pregnant women's disclosures of smoking behaviours - Pregnant women's lack of uptake of SCC [64]<br>• Lack of evidence [about its effectiveness at increasing smoking cessation rates [66]<br>• Has not been adequately tested with pregnant women [68]<br>• Low chances of success [71]<br>• Optimism of intervention effectiveness [24]	

**6. Beliefs about Consequences** (Acceptance of the truth, reality, or validity about outcomes of a behaviour in a given situation)	• Patients resistance to advice [51]<br>• Competing priorities in the visit (e.g., acute illness) [51]<br>• Provider: Beliefs: Perception that counselling could be detrimental to the patient-provider relationship [55]<br>• Advise and Assess - Fear of provoking resistance [56]<br>• Advise - More urgent priorities in prenatal care [56]<br>• Other more pressing priorities in antenatal care, especially HIV infection [57]<br>• Lack of knowledge of effectiveness of clinic-based smoking cessation interventions [57]<br>• Apparent lack of concern about smoking in pregnancy among women [57]<br>• Tensions between providing SCC and providing maternal care - Tipping the balance and risking non-attendance [64]<br>• Tensions between providing SCC and providing maternal care - Greater concerns for women's welfare than smoking status [64]<br>• Difficult conversations [65]<br>• Smoking as a social activity [65]<br>• Social and environmental context: Optimism and patient context [70]	• Beliefs about consequences [53]<br>• Doctors accept smoking should be given greater priority as preventive pregnancy risk [57]<br>• Smoking accepted as factor in current high rates of low birth weight, preterm labour, abruptio placentae [57]<br>• Doctors acknowledge that current approach to smoking cessation counselling is ineffective [57]<br>• I think providing smoking cessation support for pregnant women increases the chances that they'll quit [67]<br>• Advising women to quit smoking risks pushing them away from antenatal care – reverse scored [67]<br>• The harms of smoking in pregnancy are not as great as the other risks that women face – reverse scored [67]<br>• Talking with women about quitting smoking is a good use of my time [67]<br>• Advising women to quit smoking is one of the main things that can be done to help women have healthy babies [67]<br>• Referring women to the Quitline is an effective way of assisting pregnant women to quit [67]<br>• High priority [24]
**7. Reinforcement** (Increasing the probability of a response by arranging a dependent relationship, or contingency, between the response and a given stimulus)	• Lack of incentives [66]	•
**8. Intentions** (A conscious decision to perform a behaviour or a resolve to act in a certain way)		• I intend to provide smoking cessation support to each pregnant smoker [67]<br>• I intend to follow up with all smokers about their smoking at later visits (after the booking in visit) [67]<br>• I intend to advise all pregnant smokers to quit [67]

**9. Goals** (Mental representations of outcomes or end states that an individual wants to achieve)		• Motivation and goals [53]<br>• Doctors wish to have greater success in advising women to quit [57]<br>• After the booking in visit, providing smoking cessation support is not as important to me as providing some other aspects of antenatal care – reverse scored [67]<br>• I place a high priority on helping women quit smoking [67]
**10. Memory, attention and decision processes** (The ability to retain information, focus selectively on aspects of the environment and choose between two or more alternatives)		• Memory, attention, and decision processes [53]<br>• I always remember to advise women who smoke to quit smoking [67]
**11. Environmental context and resources** (Any circumstance of a person's situation or environment that discourages or encourages the development of skills and abilities, independence, social competence and adaptive behaviour)	• Lack of community resources for referral [51]<br>• Cost of medications [51]<br>• No or limited reimbursement for smoking cessation [51]<br>• Lack of time [51]<br>• Subtheme: Requiring visual resources [52]<br>• Subtheme: Reducing NRT cost [52]<br>• Time limitations [54]<br>• Lack of referral resources for adequately dealing with prenatal tobacco use problems once identified [54]<br>• Provider: Attitudes: Lack of motivation to intervene. Large workload, without adequate remuneration or encouraging feedback [55]<br>• Clinics: Failures in provider-provider communication and provide-director-provider communication [55]	• Environmental context and resources [53]<br>• Provider: Time in the waiting room could be used to deliver smoking cessation counselling [55]<br>• Provider: Use of written materials would facilitate counselling [55]<br>• Provider: Use of structured protocol of how to counsel women would increase providers' confidence [55]<br>• Doctors welcome prospect of receiving educational aids, guidelines and attractive media for distribution to pregnant women [57]<br>• Subsidised oral NRT on PBS [58]<br>• Medicare item number for cessation counselling [58]<br>• Improved access to NRT patches [58]

	• Clinics: Amount of time in visit is insufficient to deal with additional health services [55]<br>• Clinics: Lack of prioritisation of smoking cessation in current practice and protocols [55]<br>• Advise, Assess, Assist and Arrange - Lack of time [56]<br>• Lack of attractive educational resources to distribute to pregnant smokers [57]<br>• Current levels of stress among health care providers in the public sector [57]<br>• General despondency among staff about working conditions in the public sector [57]<br>• Acute shortage of midwives in public sector antenatal services [57]<br>• Too little time to interact with and educate pregnant women [57]<br>• Lack of smoking cessation materials to give to patients [59]<br>• Time constraints [60]<br>• Midwives – time (Barrier/ Enabler) [61]<br>• The availability of free client brochures would stimulate me to discuss quitting smoking more often [62]<br>• I miss simple tools or gadgets (e.g. infographics or apps) to trigger the conversation on smoking cessation [62]<br>• I sometimes find it difficult to discuss quitting smoking due to the patient's insufficient mastery of the Dutch language [62]<br>• I would discuss smoking cessation more often if some financial compensation was provided, for example by means of a reimbursable consultation [62]<br>• I usually have enough time to discuss quitting smoking.* % barrier reported - reverse scored [62]<br>• Lack of time [63]<br>• Organisational barriers in the delivery of SCC<br>- Lack of time, priority and continuity<br>- Need for improved tools and practices [64]<br>• Time [65]	• Systems and Resources: the Electronic Medical Record (EMR) (Barrier/ Enabler) [65]<br>• Our service has good pamphlets and resources to support pregnant smokers to quit [67]<br>• Our service has capacity to provide smoking cessation support for pregnant smokers [67]<br>• I don’t have time to provide smoking cessation support in visits after the booking in visit – reverse scored [67]

	• Lack of time [66]<br>• Lack of time [69]<br>• Social and environmental context: Time and continuity of care [70]<br>• Lack of time [71]<br>• Sufficient time [24]<br>• Sufficient resources [24]	
**12. Social influences** (Those interpersonal processes that can cause individuals to change their thoughts, feelings, or behaviours)	• Lack of patient interest [60]<br>• Midwives - Impact on relationship with client (barrier/ enabler) [61]<br>• Midwives - Expected that other priorities more important for women [61]<br>• Parents do not expect advice [71]<br>•	• Social Influences [53]<br>• Lack of behavioural regulation, reinforcement and social influence (champions highly influential) [65]<br>• Specialist support [66]<br>• GP role and identity: Patient expectations [70]<br>• GP role and identity: Building rapport [70]<br>• Feel comfortable raising the issue with a pregnant woman [24]<br>•
**13. Emotion** (A complex reaction pattern, involving experiential, behavioural, and physiological elements, by which the individual attempts to deal with a personally significant matter or event)	• Feeling uncomfortable [71]	• Emotion [53]<br>• I get satisfaction from providing smoking cessation support to pregnant women [67]<br>• I often find talking with pregnant smokers about their smoking makes me feel uncomfortable (reverse scored) [67]<br>• GP role and identity: Job role and satisfaction [70]
**14. Behavioural regulation** (Anything aimed at managing or changing objectively observed or measured actions)		• Action planning [53]
