# Supplementary material for: Barriers and enablers to addressing smoking, nutrition, alcohol consumption, physical activity and gestational weight gain (SNAP-W) as part of antenatal care: A mixed methods systematic review
Source: Implement Sci Commun. 2024 Oct 9;5:112. doi: 10.1186/s43058-024-00655-z (PMC11462853; doi:10.1186/s43058-024-00655-z)
Supplement: Supplementary file 6 — Supplementary Material 6. [file 43058_2024_655_MOESM6_ESM.pdf]

Additional File 6. Barriers and enablers reported by study authors coded to TDF- ALCOHOL

TDF Domain (definition)	Barriers	Enablers
**1. Knowledge** (An awareness of the existence of something)	• Inadequate information on referral resources [77][1]<br>• Views on the guidelines: limited awareness of source of the Guidelines [78]<br>• Views on the guidelines: Inadequate dissemination of Guidelines [78]<br>• Lack of training [80]	
**2. Skills** (An ability or proficiency acquired through practice)	• Lack of training [73]<br>• Skills [74]<br>• Difficulty in unit conversion [75]<br>• Clinician's need for additional trainings [77]	• More knowledge about conversational techniques for use when alcohol-related symptoms are evident [76]<br>•
**3. Social/professional role and identity** (A coherent set of behaviours and displayed personal qualities of an individual in a social or work setting)		• Views on the guidelines: Provision of evidence based information [78]
**4. Beliefs about capabilities** (Acceptance of the truth, reality or validity about an ability, talent or facility that a person can put to constructive use)	• Beliefs about capabilities [74] [79]	
**5. Optimism**		

[1] To find the referenced study refer to the reference list in main article.

(The confidence that things will happen for the best or that desired goals will be attained)		
**6. Beliefs about Consequences** (Acceptance of the truth, reality, or validity about outcomes of a behaviour in a given situation)	• Patient denial and resistance to treatment[72]<br>• Patient sensitivity [73] [77]<br>• Beliefs about consequences [74] [79]<br>• Low priority [75]<br>• Overload of information at booking appointments [75]<br>• Communication with women: Open conversation style with women [78]	
**7. Reinforcement** (Increasing the probability of a response by arranging a dependent relationship, or contingency, between the response and a given stimulus)		
**8. Intentions** (A conscious decision to perform a behaviour or a resolve to act in a certain way)		
**9. Goals** (Mental representations of outcomes or end states that an individual wants to achieve)		

**10. Memory, attention and decision processes** (The ability to retain information, focus selectively on aspects of the environment and choose between two or more alternatives)		
**11. Environmental context and resources** (Any circumstance of a person's situation or environment that discourages or encourages the development of skills and abilities, independence, social competence and adaptive behaviour)	• Time limitations / constraints [72] [73] [75] [78] [80]<br>• Poor resources [73]<br>• Environmental context and resources [74]<br>• Time restraint during prenatal visits [77]<br>• Unclear guidelines on prenatal alcohol used [77]<br>• Ambiguity in written informational materials [77]<br>• Issues related to language and ethnicity [80]<br>• Lack of organizational support [80]	• Improved local guidelines for how to address alcohol with expectant parents [76]<br>• Clearer national-level guidelines for work with risky drinkers [76]<br>• Clearer management-level decisions about work with risky drinkers [76]<br>• Greater possibility of more time for extra visits to midwife when<br>• identifying women with risk behaviour [76]<br>• Strategies in addressing alcohol use: Use of validated screening tool/s [78]<br>•
**12. Social influences** (Those interpersonal processes that can cause individuals to change their thoughts, feelings, or behaviours)	• Social influences [74] [79]<br>• Views on the guidelines: Differing advice being provided by health professionals [78]	• Improved opportunities of support and instructions from specialists [76]<br>• Communication with women: Establishing trusted relationships with women [78]
**13. Emotion** (A complex reaction pattern, involving	• Emotion regulation [74]	•

experiential, behavioural, and physiological elements, by which the individual attempts to deal with a personally significant matter or event)		
**14. Behavioural regulation** (Anything aimed at managing or changing objectively observed or measured actions)	• Behavioural regulation [74]	•
