# Supplementary material for: Barriers and enablers to addressing smoking, nutrition, alcohol consumption, physical activity and gestational weight gain (SNAP-W) as part of antenatal care: A mixed methods systematic review
Source: Implement Sci Commun. 2024 Oct 9;5:112. doi: 10.1186/s43058-024-00655-z (PMC11462853; doi:10.1186/s43058-024-00655-z)
Supplement: Supplementary file 7 — Supplementary Material 7. [file 43058_2024_655_MOESM7_ESM.pdf]

Additional file 7. Results of individual studies Nutrition, Physical Activity and Gestational Weight Gain

TDF Domain (definition)	Barriers	Enablers
**1. Knowledge**<br>(An awareness of the existence of something)	• Knowledge of optimal gestational weight gain [81][1]<br>• Approaches to Care - Unfamiliarity with established guidelines [83]<br>• Perceived deficiencies in the working situation - Lack of specific competence (Weight related knowledge) [85]<br>• Lack of training, knowledge and confidence [87]<br>• Unawareness of suitable resources and opportunities [87]<br>• Reliance on common sense and own experience as opposed to evidence-based practice [87]<br>• Individual level influences on practice: General knowledge of gestational weight gain, nutrition and physical activity [92]<br>• Individual level influences on practice: Detailed knowledge of practice guidelines [92]<br>• Despite low awareness of guidelines, GWG advice is provided. Sub-Theme: Knowledge of guidelines is low [97]	
**2. Skills**<br>(An ability or proficiency acquired through practice)	• Lack of nutritional instruction in medical school and other educational constraints [84]<br>• Perceived deficiencies in the working situation - Lack of specific competence (Communication skills) [85]<br>• Loss of skills [89]<br>• Clinician characteristics [90]<br>• Counselling as an opportunity: Navigating within cultural traditions [91]<br>• "I was not well trained to have these conversations" [93]<br>• Knowledge: Lack of formal training (especially OB=GYNs) [94]	• Professional development [87]<br>• Counselling as walking the thin ice: Acting upon individual needs [91]<br>• Counselling as an opportunity: Identifying women's individual enablers [91]
**3. Social/professional role and identity**<br>(A coherent set of behaviours and displayed personal qualities of an individual	• Complications with higher risk populations - litigation concerns [84]<br>• Conflicting responsibilities in midwives' professional identity - Promote healthy gestational weight gain [85]<br>• Practical barriers to management - Care received from other services [96]	• Midwives feel responsible and have got skills and experience (to discuss nutrition) [82]<br>• "I should do this more." GPs considered it their professional role to support women with weight management in pregnancy [97]

[1] To find the referenced study refer to the reference list in main article.

in a social or work setting)		• Midwives are central to healthy lifestyle education process and opportunities exist for support to promote healthy GWG - a) Key providers of lifestyle behaviour education [98]
**4. Beliefs about capabilities** (Acceptance of the truth, reality or validity about an ability, talent or facility that a person can put to constructive use)	• Confidence in providing gestational weight gain counselling [81]<br>• Referral - Variable approach by antenatal clinicians [88]<br>• Counselling as a challenge: Responding to high-achieving women [91]<br>• Counselling as a challenge: Responding to perceived barriers to physical activity in pregnant women [91]<br>• Behaviours: Use personal experiences in counselling [94]<br>• Midwives are central to healthy lifestyle education process and opportunities exist for support to promote healthy GWG - b) Lack of confidence in addressing weight and GWG [98]	
**5. Optimism** (The confidence that things will happen for the best or that desired goals will be attained)	• Attitudes and Beliefs - Scepticism about counselling's impact on patient [83]<br>• Attitudes and Beliefs - Patient more influenced by other factors [83]<br>• Patient motivation and compliance [86]<br>• Communication - Cultural Challenges [88]<br>• Perceptions and context of pregnant women - Attitudes to clinics and interventions [88]<br>• Perceptions and context of pregnant women - Lack of understanding [88]<br>• Referral - Perceived lack of usefulness of antenatal referral [88]<br>• Counselling as a challenge: Responding to the cultural tug-of-war [91]<br>• Counselling as walking the thin ice: Fearing no success [91]<br>• Attitudes: Uncertainty, doubt about counselling effectiveness [94]<br>• Working against the odds at time. Sub-Theme: Social environment [97]<br>• Optimism and reality. Women's capacity to put the advice they receive into practice is challenged by the broader physical and social environment [97]	• Optimism and reality. General Practitioners generally believe that primary care is an ideal setting to provide GWG advice and that women respond well to the advice they receive [97]

**6. Beliefs about Consequences**<br>(Acceptance of the truth, reality, or validity about outcomes of a behaviour in a given situation)	• Gestational weight gain counselling experience [81]<br>• Gestational weight gain counselling was a low priority [81]<br>• Protecting women from embarrassment [81]<br>• Nutrition is considered a precarious topic [82]<br>• Nutrition is not prioritised [82]<br>• Priority of weight gain relative to other common prenatal issues [83]<br>• Approaches to Care - Reactive approach [83]<br>• Attitudes and Beliefs - Sensitivity of topic [83]<br>• Midwives use avoidant behaviours to cope with fear of inflicting worries, shame or feelings of guilt in pregnant women (main theme) [85]<br>• Conflicting responsibilities in midwives' professional identity<br>• - Be empathic and consider weight stigma<br>• - Keep the woman calm and at ease [85]<br>• Time constraints during short antenatal visits [86]<br>• Sensitivity of topic [86]<br>• Perceptions of vulnerability relating to inherent fears and exposure to risk [87]<br>• Communication – Sensitivity [88]<br>• Attitudes to weight - Normalisation of higher BMI [88]<br>• Patient characteristics [90]<br>• Disadvantages of Routine Weighing [90]<br>• Evidence for Routine Weighing and Interventions [90]<br>• Individual level influences on practice: Priority level [92]<br>• Individual level influences on practice: Sensitivity of the discussion [92]<br>• “I think much of what we say is not that useful” [93]<br>• “You have to be very thoughtful about how to frame it” [93]<br>• Attitudes: Sensitivity of topic [94]<br>• Patients not interested in changing behaviours [95]<br>• High relapse rates for weight management behaviours [95]	• Advantages of Routine Weighing [90]<br>• Concern for physical and psychological health of pregnant women - b) Concern for the physical health of women [98]

	• Provision of advice regarding gestational weight gain and healthy lifestyle behaviour advice [96]<br>• Attitudes and practices around routine gestational weighing [96]<br>• "I should do this more." Providing GWG advice is often deprioritised in busy consultations [97]<br>• Working against the odds at time. Sub-Theme: Meeting women where they are at [97]<br>• GWG is a low priority for midwives - c) Excess GWG not seen to be common or problematic by many [98]<br>• Concern for physical and psychological health of pregnant women - a) Concern for the psychological impacts of weight discussions and women's inappropriate views on weight gain [98]	
**7. Reinforcement**<br>(Increasing the probability of a response by arranging a dependent relationship, or contingency, between the response and a given stimulus)		
**8. Intentions**<br>(A conscious decision to perform a behaviour or a resolve to act in a certain way)		
**9. Goals**<br>(Mental representations of outcomes or end states that an individual wants to achieve)		
**10. Memory, attention and decision processes**<br>(The ability to retain information, focus selectively on aspects of	• Behaviours: Reactive approach; lack of baseline assessment [94]	

the environment and choose between two or more alternatives)		
**11. Environmental context and resources** (Any circumstance of a person's situation or environment that discourages or encourages the development of skills and abilities, independence, social competence and adaptive behaviour)	• Not having enough time [81]<br>• Resources and education mainly focus on food risks [82]<br>• Approaches to Care - Lack of accessible resources [83]<br>• "We Can't Win": Institutionalized Barriers to Prenatal Nutrition Counselling [84]<br>• Time and medical school preparation constraints that structure physician-patient interactions- short visits and low continuity [84]<br>• Perceived deficiencies in the working situation -Lack of resources and support (Time, Guidelines and routines, Limited access to other professions and support groups) [85]<br>• Language barrier with immigrant populations [86]<br>• Time constraints and ensuing compromises [87]<br>• Referral - Variable success in referral to dietician/ scan [88]<br>• Referral - Transfer of care [88]<br>• Time constraints [89]<br>• Funding issues [89]<br>• Communication with Midwife Led Maternity Carers [89]<br>• Counselling as a challenge: Fighting lack of resources [91]<br>• System-level influences on practice: Time and compensation [92]<br>• System-level influences on practice: Access to allied health services [92]<br>• "You're always kind of under the time crunch" [93]<br>• "Some clients just really can't afford a lot of nutritious foods" [93]<br>• "I don't necessarily have the stuff handy" [93]<br>• Lack of time [95]<br>• Patients cannot afford referrals [95]<br>• Lack of community resources for referral of patients [95]<br>• Practical barriers to management<br>- Cost	• Reliable information sources [82]<br>• Group consultation (eg. Multidisciplinary collaboration) [82]<br>• Inter-professional collaboration [87]<br>• Communicating effectively through simple, credible resources [87]<br>• Midwifery approach - Continuity of care [88]<br>• Practical focus - Acquiring scales [88]<br>• Collaborative approach [88]<br>• Suggested improvements – Dietician [88]<br>• Suggested improvements – Guidelines [88]<br>• Systems and Resources - Available resources [90]<br>• Systems and Resources - Standardising and normalising the process [90]<br>• Systems and Resources – Documentation [90]<br>• How GPs feel they could be best supported<br>- Multidisciplinary support<br>- Electronic/written resources [96]<br>• Midwives are central to healthy lifestyle education process and opportunities exist for support to promote healthy GWG<br>- c) Support for midwives to promote healthy GWG<br>- d) Features and content of an optimal intervention to promote healthy GWG [98]<br>•

	- Space<br>- Time<br>- Organisational structure [96]<br>• Lack of everyday resources.<br>- Lack of time<br>- Lack of resources<br>- Lack of clear guidance [97]<br>• GWG is a low priority for midwives - d) Limited resources to address GWG and lifestyle behaviours [98]	
**12. Social influences** (Those interpersonal processes that can cause individuals to change their thoughts, feelings, or behaviours)	• Despite low awareness of guidelines, GWG advice is provided. Sub-Theme: Providing advice, but only to those who need it most [97]	• Counselling as walking the thin ice: Guarding the relationship [91]
**13. Emotion** (A complex reaction pattern, involving experiential, behavioural, and physiological elements, by which the individual attempts to deal with a personally significant matter or event)		
**14. Behavioural regulation** (Anything aimed at managing or changing objectively observed or measured actions)		
